# Perceived social support and discrimination and oral health behaviors in adolescents

**DOI:** 10.1002/cre2.443

**Published:** 2021-05-20

**Authors:** Maryam Amin, Christian Schumacher, Babak Bohlouli

**Affiliations:** ^1^ Division of Pediatric Dentistry University of Alberta Edmonton Alberta Canada; ^2^ Department of Dentistry University of Alberta Edmonton Alberta Canada; ^3^ Department of Emergency Medicine University of Alberta Edmonton Alberta Canada

**Keywords:** adolescent, dentistry, oral health, social support

## Abstract

**Objectives:**

The aims of this study were to examine the associations between perceived social support and oral health behaviors among adolescents and if this perception had a protective effect against the influence of perceived racial discrimination on oral health behaviors in this population.

**Material and methods:**

Participants of this cross‐sectional study were adolescents aged 12–18 years recruited from University dental clinic. They completed a questionnaire comprising three sections: demographics (14 items), oral health behaviors (6 items), and validated Personal Resource Questionnaire (25 items). Perceived discrimination was evaluated by a question asking if the adolescent had ever experienced discrimination based on their race.

**Results:**

Of 252 participants, mean (SD) age of 14 (1.8) years, 60% were girls, 56% were self‐identified as White, and 81% were born in Canada. Discrimination was reported by 21%. Frequency of toothbrushing and self‐rated oral health were significantly associated with increased levels of perceived social support. Sugar consumption was significantly different for participants with and without perceived racial discrimination (*p*‐value = 0.002). Perceived social support did not act as a buffer against perceived racial discrimination for sugar consumption (OR = 1.00; 95% CI: 0.98–1.01).

**Conclusions:**

Adolescents' perceived social support affected some aspects of their oral health but did not moderate the influence of perceived racial discrimination.

## 1 INTRODUCTION

Oral health contributes greatly to adolescents' overall health (U.S. Department of Health and Human Services, 2000). Despite the importance of oral health, dental care is the most unmet health care need of children and adolescents (Newacheck et al., 2000). Centers for Disease Control and Prevention outlined that dental caries is among the most common chronic diseases in children worldwide (Hygiene‐related diseases, n.d.). Adolescents can manage dental caries by following certain guidelines: ensuring adequate fluoride intake, daily brushing and flossing of teeth, lowering intake of cariogenic carbohydrates, having periodic dental examinations, and having sealants and restorative work completed when needed (American Academy of Pediatric Dentistry, n.d.). Despite these recommendations, adolescence is still associated with heightened levels of caries and periodontal disease due to increased intake of cariogenic carbohydrates and ignoring oral health regimes (American Academy of Pediatric Dentistry, n.d.). Intake of sugar‐sweetened beverages (SSBs) in adolescents was positively associated with a variety of factors including fast food consumption, time spent watching TV, eating while watching TV, availability of SSBs at home, parent fast food intake, and parent and friend SSB intake (Watts et al., 2018). Adolescent toothbrushing has also been found to be multifactorial depending on lifestyle (wake‐up time, breakfast, time going to bed) and social behavior (Macgregor et al., 1996). The complex nature of adolescents' oral health makes it difficult to develop measures to improve this aspect of their lives.

Adolescent health in general has social influencers coming from family, community, national, and personal levels (Viner et al., 2012). Similarly, oral health of adolescents is also affected at the social level (American Academy of Pediatric Dentistry, n.d.). Higher family income, greater social support, and having more social networks are some social factors associated with improved oral health in adolescents (Vettore et al., 2019). Social support could be received or perceived (Dahlan et al., 2019). Received social support is made up by the social resources provided to an individual both formally (e.g., from school) or informally (e.g., from the family) (Dahlan et al., 2019). Perceived social support (PSS) on the other hand is the makeup of all of the social resources that individuals believe are accessible to them (Dahlan et al., 2019). There have been few studies exploring the effects of social support on adolescents' oral health. A positive association has been reported between caregiver's social support (received and perceived) and adolescent oral health (Iida & Rozier, 2013; Kruger et al., 2015). Mothers' PSS was positively associated with adolescents' use of dental services (Iida & Rozier, 2013), and caregivers' lower social support resulted in lower usage of dental services by adolescents (Kruger et al., 2015). Neighborhood social support and family social support have also been positively associated with parent‐reported oral health of adolescents (Reynolds et al., 2015), less gingival bleeding among 12 year olds (Tomazoni et al., 2017), and lower dental injuries in adolescents (Pattussi et al., 2006).

There are also some negative social influences having the ability to cause stress, disease, and even death (Sachser et al., 2011). One of these social stressors is perceived discrimination (PD) (Schmitt et al., 2014). Adolescents experience PD when they believe that they are treated prejudicially due to characteristics such as gender, age, ethnicity, or sexuality (Skosireva et al., 2014). In adolescents, PD has been associated with psychological problems such as anxiety, depression, self‐esteem issues, and psychological distress (Schmitt et al., 2014). To the best of our knowledge, there are no known studies on the effect of PD on adolescent oral health. In addition, despite the many studies reporting how social support may buffer the effects of stress on health and well‐being (Thoits, 2011), not much has been done in the oral health domain especially among adolescents. Therefore, the aims of this study were to (a) examine the association between PSS and oral health behaviors among adolescents and (b) determine if the PSS protects against the influence of perceived racial discrimination (PRD) on oral health behaviors.

## 2 MATERIAL AND METHODS

### 2.1 Study design

This cross‐sectional study was conducted in adherence to the Helsinki Declaration and results were reported based on the STROBE guidelines. The study protocol was approved by the University of Alberta Research Ethics Board (Protocol # Pro00077682).

### 2.2 Setting and participants

A convenience sample of adolescents 12–18 years old were recruited from the University of Alberta dental clinic for this cross‐sectional study. The inclusion criteria involved the ability to read and comprehend English and the absence of a history of significant physical or mental disabilities.

### 2.3 Data collection/procedure

A research assistant described the study to patients and their parents while waiting for their appointment in the dental clinic. They received an information letter, and those who accepted to participate in the survey completed the informed consent form. Once written consent and assent forms were taken, the participants were asked to complete a questionnaire consisting of three sections. There were no missing data in questions related to any of the three sections in the cohort of study.

### 2.4 Outcome variables

In this cross‐sectional study, four oral health‐related behaviors of adolescents were allocated as outcome variables: frequency of sugary food or sugary drink intake (never or < once a day, ≥ once a day); frequency of brushing (< twice a day, ≥ twice a day); last dental visit (within the last 12 months, over 1 year or never had one); and pattern of dental visit (regular check‐up, non‐urgent or urgent dental problems). One question also measured self‐reported oral health of the participants.

### 2.5 Independent variables

Participants' PSS was investigated using the validated Personal Resource Questionnaire (PRQ85) (Weinert, 1987). This scale contains 25 items with five dimensions of support measured on a Likert scale of 1–7. In our study, participants' response was measured on a Likert scale of 1–5 (1 = strongly disagree to 5 = strongly agree). This change was made with permission. The PRQ85 score was determined by adding the numbers. Scores from items 4, 7, 10, 16, 24 were reversed (5 = 1, 4 = 2, 3 = 3, 2 = 4, 1 = 5). Higher scores indicated higher levels of PSS (Weinert, 1987). PRD was also evaluated based on one question: “Have you ever been treated unfairly or discriminated against based on your race?” with the options of “Yes” or “No.”

### 2.6 Covariates

The covariates included: children's gender (male or female) and age (between 12 and 18 years old), children's ethnic background and place of birth (born in Canada: yes or no), number of children in the family, mothers' level of education (high school/under, college/university), monthly household income ([Canadian Dollar] less than $2000, $2000–$4000, more than $4000), and dental insurance (yes or no).

### 2.7 Data analysis

Continuous variables were described using means, standard deviations, and ranges while categorical variables were described with percentages as appropriate. To define the effect of the demographic factors on the PRQ85 score, univariate analyses were performed. Student's *t*‐test was used to find PRQ85 score differences in categories of oral health behaviors. Chi‐square test was conducted to explore statistical proportion differences of oral health outcomes in participants with and without PRD. Adjusted logistic regression model was conducted to examine association of oral health behavior with PRQ85 scores. Cronbach's alpha values were calculated to report internal consistency among items of study in the respective scales.

Moderated models of regression analyses were used to examine the hypothesis that the association between PRD and oral health behaviors occurs primarily under certain conditions with PSS serving either as an amplifier or as a buffer (Warner, 2008). Moderation was examined by constructing one hierarchical regression equation that included PRD, PRQ85, and a multiplicative term representing the interaction between the PRD and PSS. Models presenting a significant interaction term with a negative beta would suggest that PSS is buffering the relationship between PRD and oral health behaviors. A significant interaction term with a positive beta would indicate that PSS is amplifying the relationship (Aiken & West, 1991; Warner, 2008).

Statistical Package for Social Sciences (SPSS for Windows, version 24.0; SPSS Inc., Chicago, IL) program and results were judged to be statistically significant using 95% confidence intervals and *p*‐values less than 0.05.

## 3 RESULTS

Cronbach's alpha, a measure of internal consistency, of the PRQ85 algorithm to measure PSS was high and satisfactory (reliability coefficient = 0.90). Cronbach's alpha was greater than 0.80 and suggested that the scales were homogenous.

### 3.1 Descriptive analyses

In this study 252 participants were recruited with mean (SD) age of 14 (1.8) years old. Girls comprised 60% of participants, and there was no statistical age difference between boys and girls (*p*‐value > 0.05). Fifty‐six percentage were self‐identified as White (Caucasian), and 81% of participants were born in Canada. When responded, 65% of mothers had a college or university education, and 61% had a dental insurance plan. About 30% of participants reported their self‐rated oral health as not good. PSS scores were measured, and the mean and range were 101 and 55 to 124 respectively. PRD was reported in 52 (21%) of participants. Participants' demographics are presented in Table 1.

**TABLE 1:** Participant characteristics (*N* = 252)

Family	
Characteristics	*N* (%)
No of children in family	
1	18 (7)
2	100 (40)
≥3	134 (53)
Mother's level of education	
High school or lower	63 (25)
College/university	165 (65)
Monthly income level	
Less than $1999	7 (3)
$2000–$3999	38 (15)
More than $4000	85 (34)

Children	
Child gender	
Female	152 (60)
Child age mean (SD, range)	14 (1.77) (12–18)
Child birth place	
Canada	203 (81)
Out of Canada	49 (19)
Living with	
Single parent	60 (24)
Both parents	192 (76)
Ethnicity	
Others	110 (44)
White	142 (56)
Child dental insurance	
No insurance	56 (22)
Has insurance	154 (61)
Perceived discrimination	
Positive response	52 (21)
PRQ85	
Mean (SD, range)	101 (13.7) (55–124)

### 3.2 Outcome variables and PRQ85 mean score

PSS was significantly higher in participants who brushed their teeth twice or more per day compared to those who brushed less than twice per day (*p*‐value = 0.01). Participants who rated their oral health condition as good also had a significantly higher PRQ85 score than those who rated their oral health as poor (*p*‐value = 0.01). The point estimate of PRQ85 mean scores was different in utilization of dental services and diet. However, the differences were not statistically significant (Table 2).

**TABLE 2:** Social support (PRQ85) means score difference by Oral health behavior

Outcome variables	*N* (%)	PRQ score	*p*‐value
*Frequency of dental brushing*			
<2x/day	77 (30)	98	0.01
≥2x/day	175 (70)	102	
*Sugar‐intake frequency*			
<1x/day	130 (52)	102	0.47
≥1x/day	122 (48)	101	
*Utilization of dental services (last year)*			
Yes	215 (85)	102	0.21
No	37 (15)	99	
*Pattern of dental attendance*			
Dental problem	17 (6.7)	102	0.79
Regular check up	198 (78.5)	101	
*Self‐rated dental health*			
Fair or poor	73 (29)	98	0.01
Good	179 (71)	102	

### 3.3 Participants' demographics and PRQ85 mean score

To explore the effect of demographics on participants' PRQ85 score and consequently on their oral health behaviors, univariate analyses were conducted. A significant increase was found in the PRQ85 score of those participants living with both parents, a higher household income, and a better self‐rated oral health condition (B = 6.76, 95% CI: 2.84–10.67; B = 3.72, 95% CI: −0.24–7.68; and B = 4.48, 95% CI: 0.76–8.19 respectively). Demographics univariate analyses are presented in Table 3.

**TABLE 3:** Univariate analyses of PRQ85 with demographics

	Demographics	Beta coefficient	95% CI
Social support score (PRQ85)	Male	−2.54	−6.01–0.92
	Born in Canada	0.10	−4.20–4.41
	Age	−0.31	−1.27–0.64
	Ethnicity: White	0.88	−2.55–4.32
	Living with both parents^a^	6.76	2.84–10.67
	Mother university education	−0.78	−4.64–3.07
	Number of children	1.15	1.56–3.88
	Income^a^	3.72	−0.24–7.68
	Have dental coverage	−0.59	−4.72–3.52
	Self‐rated oral health^a^	4.48	0.76–8.19

^a^ Significant.

### 3.4 Regression model: PRQ85 score and oral health behavior

In adjusted logistic regression analyses to participants' living status and household income, we found that the likelihood of toothbrushing twice per day or more increased 3% by any unit increase in PRQ85 score (OR = 1.03; 95% CI: 1.01–1.05). Risk of sugar consumption also decreased with higher PSS: any unit increase in PRQ85 score lowered the risk of sugar consumption by 3% (OR = 0.97; 95% CI: 0.94–0.99). PRQ85 score and oral health behavior regression analyses data can be found in Table 4.

**TABLE 4:** Adjusted odds ratio of Oral health behavior with PRQ85; multivariate analyses

	Odds ratio	Z	95% CI
Frequency of dental brushing	1.03	2.17	1.01–1.05
Sugar‐intake frequency	0.97	−2.06	0.94–0.99
Utilization of dental services (last year)	1.01	0.89	0.98–1.04
Pattern of dental attendance	0.98	−1.18	0.95–1.02
Self‐rated dental health	1.04	2.56	1.00–1.57

### 3.5 Moderation test

Moderation analyses were performed to examine the impact of PSS on the association between PRD and oral health behaviors. Study findings showed that among the three oral health behaviors, only sugar consumption was statistically different in participants with and without PRD (χ^2^ = 9.37, *p*‐value = 0.002). In univariate analyses, risk of sugar consumption with meal was 2.67 times in those participants with the experience of PRD. (OR = 2.67; 95% CI: 1.40–5.08). Moderation analyses of PSS on the association of PRD and sugar consumption did not support our hypothesis, and there was no buffering effect of PSS on the association. Odds ratio of the interaction term of PRD and PSS was not significant in the model of hierarchical regression Equations. (OR = 1.00; 95% CI: 0.98–1.01).

## 4 DISCUSSION

The main focus of this study was to examine the effects of adolescents' perceived social support on their oral health behaviors. In addition, we hypothesized that the perceived social support could have a protective influence on the negative outcomes of perceived racial discrimination in adolescents. Among the five oral health outcomes, frequency of toothbrushing and self‐rated oral health were positively associated with increased levels of PSS. For the other three oral health variables (sugar intake, utilization of dental services, and reason for dental visit), there was no significant association to PSS. These results moderately support the idea that increased levels of PSS are associated with improved oral health and follow a similar trend to how other types of social support including received social support from neighborhood, caregivers, or family and PSS of caregivers affect adolescents' oral health (Iida & Rozier, 2013; Kruger et al., 2015; Pattussi et al., 2006; Reynolds et al., 2015; Tomazoni et al., 2017).

Our results suggest that PSS could affect various aspects of adolescent oral health differently. Adolescents with higher perceptions of social support were inclined to brush their teeth more often in our study. Similarly, findings from a previous study suggested that family social support had a positive effect on toothbrushing of adolescents (Furuta et al., 2012). Perhaps, adolescents who receive or perceive more social support from their family, spend more time around them resulting in receiving encouragement from them to practice oral health regimens. Financial reasons may also play a role in this relationship since adolescents that come from families with a higher income would naturally have more access to oral hygiene tools such as toothbrushes, toothpaste, and floss. From previous literature, higher socioeconomic status of the family has been associated with a greater toothbrushing frequency in adolescents (Gomes et al., 2020).

In our study, sugar intake and utilization of dental services were not significantly associated with PSS even though a positive trend was observed between these two variables and PSS. Evidence of associations between social support and dietary habits among adolescents is mixed (Anderson Steeves et al., 2016). Although there is a study that supports the idea that social support has a positive effect on dietary habits of adolescents (Di Noia & Byrd‐Bredbenner, 2013), another study was not able to find an association between the two variables (Anderson Steeves et al., 2016). A reason for this inconsistency is not known at this time. Perhaps, there are other factors outside of the social realm that have a greater impact on the diet of adolescents (Anderson Steeves et al., 2016).

For the usage and pattern of dental services, since parents are still in charge of taking their adolescents to the dentist, it was expected not to find a correlation between adolescent's PSS and these outcomes. For this reason, studying dental utilization among adolescents is better suited for research on adolescents' oral health in the context of parent or caregiver social support, and results from these studies have found a positive relationship between caregiver/parent social support and dental utilization by adolescents (Iida & Rozier, 2013; Kruger et al., 2015).

Demographically, the adolescents living with two parents and having a higher household income had significantly higher levels of PSS. Despite little evidence exploring the relationship between adolescent demographics and their PSS, it is logical that these two demographics would be associated with higher perceived social support in adolescents. Adolescents with two parents have more caregivers to rely on than adolescents with a single parent. A previous study noted that single mothers' levels of PSS were significantly lower than that of married mothers (Cairney et al., 2003). Although it has not been tested, there is the potential that a mother's PSS translates to adolescent PSS. Further, adolescents with a higher household income would naturally have more accessibility to resources that come at a cost.

In our study, we also examined the moderating effect of PSS on PRD in the context of adolescent oral health. Statistically, the results showed that sugar consumption was significantly higher in adolescents with experience of racial discrimination. These results are consistent with a previous study reporting that PRD was positively associated with unhealthy eating (Brodish et al., 2011). Unhealthy behaviors such as eating comfort food have been known to be stress alleviators (Jackson et al., 2010), which could be a reason for this association. However, our statistical analysis revealed that PSS had no significant buffering effect on the PRD. The failure of PSS as a buffer against PRD is a curious one. Perhaps, the PRD experienced by the adolescents was not high enough for PSS to have any effect as a buffer. A previous studied noted that the effectiveness of social support as a buffer for PD depends on the amount of PD experienced (Pascoe & Smart, 2009). Nonetheless, the association between PRD and sugar intake does not only provide insight into the consequence of racial discrimination in adolescent oral health (American Academy of Pediatric Dentistry, n.d.), but a high‐sugar diet is also a risk factor for obesity, cardiovascular disease, and type 2 diabetes (Fidler Mis et al., 2017).

Naturally, the validity of the results should only be interpreted considering the limitations of the study. Although the reliability coefficient of the data was calculated to be high, there are still some shortfalls of this study. For instance, there is some selection bias with the participants in the study. All of the participants were recruited at the University of Alberta dental clinic, which may have had some effect on adolescent oral health outcomes such as utilization of dental services. Another limitation of this study was the large age group considered. Adolescents aged 12 may have dissimilar views than older adolescents aged 17 and 18 having the ability to skew the results.

Our results showed that PSS had a positive association with two aspects of adolescent oral health: toothbrushing frequency and self‐rated oral health. More research is needed to focus on other aspects of oral health to determine a clearer picture of how PSS affects adolescent oral health as a whole. PRD was positively associated with higher sugar consumption. However, PSS did not have any moderating effect on this association. Once more solid conclusions have been made, policy makers and stakeholders may be able to concentrate social support programs in areas that have a greater influence on adolescent oral health. Our results also provide modest support that social resources should be concentrated in homes with single parents, lower income, and the presence of lower self‐perceptions of adolescent oral health in order to improve oral health outcomes of adolescents. Once there are more solid conclusions from larger reviews and meta‐analyses, there should be a more coherent understanding for stakeholders and policy makers. The aspirations for this growing body of literature is that adolescents' oral health and overall health will increase as a result.

## CONFLICT OF INTEREST

The authors have no conflicts of interest to disclose.

## AUTHOR CONTRIBUTIONS

Maryam Amin conceived the idea, designed the study, supervised the data collection, contributed to data analysis and interpretation of the results and writing the manuscript; Babak Bohlouli performed data analysis and contributed to interpretation of the results and reviewed the manuscript; Christian Schumacher contributed to interpretation of the results and drafted the manuscript.

## Data Availability

The data that support the findings of this study are available on request from the corresponding author. The data are not publicly available due to privacy or ethical restrictions.
